# Detection of HBV and HCV Seroprevalence using rapid point-of-care tests among earthquake survivors in Southeastern Türkiye

**DOI:** 10.1186/s12879-025-12151-3

**Published:** 2025-12-24

**Authors:** Muhammed Bekçibaşı, Derya Çağlayan, Medine Erkan, Murat Can, Şafak Kaya

**Affiliations:** 1 Department of Infectious Diseases, University of Health Sciences Gazi Yaşargil Training and Research Hospital, Diyarbakır, Türkiye; 2 Department of Epidemiology, Diyarbakır Provincial Health Directorate, Diyarbakır, Türkiye; 3 Department of Nursing, Atatürk Faculty of Health Sciences, Dicle University, Diyarbakır, Türkiye; 4 Public Health Services, Diyarbakır Provincial Health Directorate, Diyarbakır, Türkiye

**Keywords:** HBV, HBsAg, Anti-HCV, Point-of-care tests, Prevalence

## Abstract

**Background:**

Türkiye has a moderate seroprevalence of HBV infection in its general population, with the Southeastern Anatolia Region exhibiting the highest rate. In contrast, the seroprevalence of HCV infection in Türkiye is low. The study aimed to determine the prevalence of HBV and HCV in the communities affected by the earthquake in Southeastern Türkiye.

**Methods:**

This was a cross-sectional study in Southeastern Türkiye in December 2023, eleven months after the 2023 Türkiye-Syria earthquakes. HBsAg and anti-HCV were investigated using rapid screening tests on blood samples from participants selected from 251 containers, where 1,213 people resided.

**Results:**

Out of 251 samples tested, 13 (5.2%) were positive for HBV, while none (0.0%) tested positive for HCV. When comparing the risk factors for HBV infection between the positive and negative groups, earthquake survivors who tested positive for HBsAg were significantly more likely to be male sex (*p* = 0.018) and to have a family history of HBV (*p* < 0.001). There were no significant differences (*p* > 0.05) in age, number of household members, education level, marital status, history of surgery or dental procedure, tattoo-piercing, and history of blood transfusion.

**Conclusions:**

In Southeastern Türkiye, 5.2% of earthquake survivors tested positive for HBsAg using rapid point-of-care tests. More comprehensive studies are needed to determine the prevalence of HBsAg and anti-HCV in the general population and to identify risk factors.

**Clinical trial:**

Not applicable.

**Supplementary Information:**

The online version contains supplementary material available at 10.1186/s12879-025-12151-3.

## Introduction

Earthquakes are unpredictable natural disasters that can devastate people and structures globally [1]. Located in the Mediterranean’s highly active earthquake zone, Türkiye is frequently affected by severe earthquakes [2]. On February 6 and 7, 2023, two major earthquakes measuring 7.8 and 7.6 on the Richter scale occurred in Southern Türkiye, affecting 11 provinces in the Mediterranean, Eastern, and Southeastern Anatolia Region [3]. These two earthquakes and subsequent aftershocks impacted around nine million people, evacuating three million individuals from their homes and resulting in 51,000 deaths and over 100,000 injuries [4]. Following two earthquakes, the impacts of which were also felt in Diyarbakır, one of the largest provinces in the Southeastern Anatolia Region, 8,602 buildings were severely damaged. Four hundred fourteen people lost their lives, and 902 people were rescued from under the rubble [5]. The central government established temporary shelters for 2,387 earthquake survivors in the Kayapınar district using 619 containers [6].

The issues of unscreened blood transfusions, unsterilized equipment, and a lack of awareness related to earthquakes may aggravate the transmission and prevalence of viral infections in earthquake-affected communities [7].

Türkiye has a moderate (4%) seroprevalence of HBV infection in the general population, with the Southeastern Anatolia Region having the highest rate at 7.3%. Due to this regional difference, which is not seen in HCV infection, HBV endemicity is thought to be affected by crowded family structures, intra-family transmission, and poor hygiene conditions [8].

In Türkiye, the national HBV vaccination program was launched in 1998, followed by adolescent vaccination campaigns in schools between 2005 and 2009. The Ministry of Health offers free vaccinations to healthcare workers and adults in high-risk groups. As a result of these practices, the number of acute HBV cases in Türkiye has declined over the years, and there has been a significant reduction in chronic HBV infections [9].

Studies on HBV seroprevalence in the Southeastern Anatolia Region are scarce and have primarily been conducted on blood donors. Studies of blood donors in the Diyarbakır region reported that the hepatitis B surface antigen (HBsAg) positivity rate decreased from 3.14% to 1.73% due to HBV vaccination [10, 11].

The seroprevalence of HCV infection in Türkiye is low, ranging from 0.5% to 1% [12]. The seroprevalence of HCV is highest in older age groups because many were infected during periods when sterilization rules were not followed in past health practices [8]. Thus, people who received blood or blood products or had an organ transplant before 1996 in Türkiye are considered to be in the high-risk group for HCV. However, it is estimated that approximately 40% of people who inject drugs in Türkiye are anti-HCV positive, and the majority of new HCV cases are from this risk group [12].

Rapid diagnostic tests (RDT) are single-use disposable assays designed for easy use. They require no additional reagents other than those included in the test kit. These tests can be read visually and provide a simple qualitative result in under 30 min. Because of their simplicity, low cost, and quick turnaround time, they can be performed by trained lay providers or healthcare workers without the need for venipuncture to collect specimens. RDTs are especially useful in situations where standard laboratory testing services are unavailable or difficult to access [13]. Rapid point-of-care tests are crucial for screening large populations, but they must exhibit high diagnostic sensitivity and specificity [14].

While public and civil organizations offered basic health services in temporary communal living areas after the earthquake, we viewed the current situation as an opportunity for HBV and HCV screening. In this study, we aimed to evaluate the prevalence of HBV and HCV infections in earthquake survivors residing in a container city using rapid point-of-care tests.

## Materials and methods

### Study population and survey design

This was a cross-sectional study, and the samples were collected in December 2023, 11 months after the 2023 Türkiye-Syria earthquakes. The study’s inclusion criteria required participants to be at least 18 years old, reside in a container city, and be willing to volunteer. Individuals who were visitors, lacked the cognitive ability to respond to the study questions, or refused to provide their contact details were excluded from participation.

As of 2023, the population of Diyarbakır is 1,818,133, the 12th largest city in Türkiye. The population aged 18 and over is 1,146,717 [15].

The prevalence of HBV infection in our country is reported as 4%. In different studies conducted in our region, the prevalence varies between 7% and 13% [8, 16]. The number of people living in the container city is 2387. Based on these studies, the population size for HBV infection was calculated as 2387, prevalence was 7%, absolute precision was 3%, design effect was taken as 1, and the sample size was calculated as 249 at a 95% confidence level (OpenEpi: Open Source Epidemiologic Statistics for Public Health, Version).

The prevalence of HCV infection in our country is reported as 1%, while studies in our region indicate a rate of 0.7% to 2.2% [8, 16]. Accordingly, the population size for HCV infection was calculated as 2387, the prevalence was 1%, the absolute precision from prevalence was 1%, the design effect was 1, and the sample size was 329 at a 95% confidence level (OpenEpi: www.OpenEpi.com).

The study volunteers were from the earthquake-affected Diyarbakır region, which includes the towns of Bağlar, Kayapınar, and Sur. Government and non-governmental organizations provided housing, food, education, and medical care. The sample population included unemployed people, students, teachers, engineers, and workers. One person from each container was selected as a volunteer. HBsAg and anti-HCV were investigated with rapid screening tests in blood samples from volunteers selected from 251 containers representing 1213 populations.

### Specimen collection

The study utilized rapid point-of-care tests provided by the Public Health Services Directorate of the Diyarbakır Provincial Health Directorate. Earthquake survivors who agreed to undergo HBsAg and anti-HCV testing and met the eligibility criteria were included in the study. Rapid point-of-care tests were applied to volunteers who completed the questionnaire adapted from the TURHEP study [8]. Volunteers were screened for HBV and HCV using the TOYO rapid tests (Toyo HBsAg; *sensitivity*: 100% *specificity*: 99.89%, and anti-HCV test; *sensitivity*: 100% *specificity*: 100%, TurkLab, Türkiye), which are rapid, immunochromatographic assays for qualitative detection [17]. TOYO rapid test kits have been reported to perform well in predicting HBV and HCV infections in general and can be used in those in high-risk groups [18]. The test kits used in this study meet the ASSURED criteria established by the WHO for point-of-care tests (POCTs) intended for use at level 1 health centers. These criteria are: Affordable, Sensitive, Specific, User-friendly, Rapid, Robust, Equipment-free, and Deliverable to end-users [19].

Blood samples were collected from the fingertip using a lancet and recorded as either positive or negative after a 15-minute wait. Blood samples (8–10 mL) were collected from all volunteers who tested positive in the rapid tests for confirmation at the reference hospital. The tests included anti-HCV, HCV-RNA, HBsAg, anti-HBs, total anti-HBc, HBeAg, anti-HBe, HBV-DNA, and anti-HDV. Serological tests were performed using Architect i2000 SR (Abbott Laboratories, IL, USA). HBV DNA levels were measured using the COBAS AmpliPrep Total Nucleic Acid Isolation Kit (Roche Molecular Systems Inc., Branchburg, NJ, USA).

### Statistical analyses

Differences in demographic characteristics and risk factors between the positive and negative groups were analyzed using a Fisher’s Exact test. Numerical data was analyzed using a Student t-test. All analyses were performed in IBM SPSS Statistics version 30. The statistical significance level was *p* < 0.05 for all the analyses.

### Ethical consideration

This study was approved by the Clinical Research Ethics Committee of the University of Health Sciences Türkiye, Gazi Yaşargil Training and Research Hospital, on 04 August 2023, under the number 514.

## Results

Out of 619 containers, around 400 were full, and 251 earthquake survivors who resided in these containers participated in the study. Of these, 190 (75.7%) were females and 61 (24.3%) were males (Table 1). No earthquake survivors in the study tested positive for anti-HCV. Thirteen (5.2%) of the 251 earthquake survivors tested using rapid tests had HBsAg positive, confirmed with further examinations. The mean age in the HBsAg positive group was 45.8 ± 8.7 (29–64); in the HBsAg negative group, it was 45.2 ± 14.1 (22–86). When comparing the risk factors for HBV infection between the positive and negative groups, earthquake survivors who tested positive for HBsAg were significantly more likely to be male sex (*p* = 0.018) and to have a family history of HBV (*p* < 0.001). There were no significant differences (*p* > 0.05) in age, number of household members, education level, marital status, history of surgery or dental procedure, tattoo-piercing, and history of blood transfusion (Table 2).

**Table 1:** Distribution of participants’ sociodemographic characteristics

	Participants n (%)
**Age (Years)**	
22–30	31 (12.3)
31–40	74 (29.5)
41–50	71 (28.3)
>50	75 (29.9)
**Gender**	
Male	61 (24.3)
Female	190 (75.7)
**Number of household members**	
1–3	56 (22.3)
4–6	151 (60.2)
>6	44 (17.5)
**Education status**	
None	95 (37.8)
Primary school	94 (37.4)
Secondary school	22 (8.8)
High school	24 (9.6)
University	16 (6.4)
**Marital status**	
Single	26 (10.4%)
Married	225 (89.6%)
**Family history of HBV**	
Yes	36 (14.3)
No	215 (85.7)
**History of surgery**	
Yes	140 (55.8)
No	111 (44.2)
**History of dental procedure**	
Yes	129 (51.4)
No	122 (48.6)
**Tattoo or piercing**	
Yes	14 (5.6)
No	237 (94.4)
**Blood transfusion before 1996**	
Yes	11 (4.4)
No	240 (95.6)
**Intravenous drug use**	
Yes	0 (0)
No	251 (100)

**Table 2:** Characteristics of the study participants by HBsAg status

Characteristics	Participants N=251 (%)	HBsAg (-) (n=238)	HBsAg (+) (n=13)	*P* value
Age, years, mean(SD)*	45.2 ± 13.9	45.2 ± 14.1	45.8 ± 8.7	.822
Number of household members*	4.8 ± 1.9	4.8 ± 1.9	4.9 ± 1.3	.859
Less than secondary school	184 (73.3 %)	175 (73.5 %)	9 (69.2 %)	.474
Sex, male	61 (24.3 %)	54 (22.7 %)	7 (53.8 %)	.018
Married	225 (89.6 %)	213 (89.5 %)	12 (92.3 %)	.601
Family history of HBV	36 (14.3 %)	27 (11.3 %)	9 (69.2 %)	<.001
History of surgery	140 (55.8 %)	135 (56.7 %)	5 (38.5 %)	.197
History of dental procedure	129 (51.4 %)	122 (51.3 %)	7 (53.8 %)	.856
Tattoo or piercing	14 (5.6 %)	14 (5.9 %)	0	.465
Blood transfusion before 1996	11 (4.4 %)	11 (4.6 %)	0	.550

*The student’s t-test. The Fisher’s Exact test was utilized for all other statistics

**ELISA and other serological tests confirmed all positive cases from point-of-care tests

## Discussion

To our knowledge, this is the first cross-sectional study to assess the seroprevalence of HBsAg and anti-HCV using rapid point-of-care tests in the general population of Diyarbakır, Türkiye. The results of our study indicate that 5.2% of the earthquake survivors tested positive for HBsAg. Anti-HCV was not detected positive in any of the tests. Additionally, being male and having a family history of HBV were identified as risk factors in individuals who tested positive for HBsAg in the study population.

Studies conducted on the general population in Türkiye have identified certain risk factors for HBV infection. These include close contact with an infected patient, hospitalization, living in the Southeastern region, being male, being married, having a low education level, undergoing dental interventions, and having a history of using non-disposable syringes [8, 20, 21]. Whereas, our study identified male gender and a family history of HBV as statistically significant risk factors.

Thanks to the national HBV vaccination program, which started in 1998, the positivity rate of HBsAg among children, adolescents, and young adults in Türkiye has declined significantly. With the decrease in acute hepatitis B cases, the disease has begun to be seen in older age groups [22]. In a recently published multicenter study covering the years 2005–2019, it was reported that the HBsAg positivity rate in pregnant women born after HBV vaccination was initiated was meager, and 97% of HBsAg-positive women were born before 1998 [9]. Consistent with the existing data, among HBsAg positive cases (age range: 29–64) in our study, no earthquake survivors were born after 1998.

In Türkiye, one in three adults over the age of 18 has been exposed to HBV, and it is estimated that more than 2 million (4%) adults are HBsAg positive. However, only about 12% of these individuals know their condition [8]. While HBsAg positivity has decreased over the years in Türkiye, sexual and horizontal transmission remain significant, particularly among young adults. The eastern and southeastern regions, where the young population and fertility rate are high, rank first in the Hbsag positivity rate. This issue arises primarily from failing to identify HbsAg-positive women during pregnancy and the insufficient immunization provided to newborns at birth [23]. There are limited studies on the seroprevalence of HBV in our region. In a 2003 study of the general population, the HBsAg positivity rate was 8.2% in rural areas and 6.2% in urban areas [21]. The prevalence of HBsAg was found to be between 9 and 15.9% in patients treated as inpatients and outpatients with various diagnoses at Diyarbakır Training and Research Hospital between 2005 and 2012 [16]. This study’s 5.2% positivity rate indicates that while HBsAg positivity in the Diyarbakır region has decreased over the years with HBV vaccination, it remains above the national average.

In Türkiye, HCV infection is more prevalent among vulnerable groups and has an estimated prevalence of 0.5-1% in the general population [8, 24]. Individuals who inject drugs and those in prison represent the majority of new HCV cases [12]. In the region’s population-based prevalence study, the anti-HCV positivity rate was 0.6%. The authors concluded that the lack of significant risk factors contributing to transmission and the small number of vulnerable groups in society were the main reasons for the low prevalence [25].

The research by Khan et al. in 2005 on the prevalence of HCV and HIV infections in areas impacted by the earthquake in Pakistan indicated that a higher prevalence of HCV was recorded 11 months after the disaster. However, this increase was not statistically significant [7]. Contrary to this study, we did not find any anti-HCV-positive samples. This result may be explained by the absence of intravenous drug addiction among the earthquake survivors and the insufficient number of available samples.

We demonstrated the use of rapid testing among individuals living in challenging conditions after the earthquake as an opportunity to identify patients infected with HBV and HCV. As a suggestion, these implementations could be carried out in larger populations, coordinated by the government and other civil society organizations.

Our study has several limitations. First, while laboratory verification was conducted for positive samples, negative samples could not be checked due to the costs. Users’ errors during rapid test implementation and analysis may have impacted results. Second, since the study focused on earthquake survivors with housing issues, there may be selection bias toward lower socioeconomic communities. Third, our study did not detect HCV infections, likely because the target population size was not reached. Since participation was voluntary, some residents of the container chose not to take part in the study, while others could not be contacted. A more extensive analysis may be necessary to understand the dynamics of HCV in these regions accurately. Lastly, individuals aware of their HBV or HCV infection might avoid study participation due to privacy concerns, as they may not wish for their condition to be disclosed.

## Conclusion

This study indicates that periods following natural disasters like earthquakes allow community screening using rapid point-of-care tests, particularly given the challenges in conducting HBV and HCV infection prevalence studies in Türkiye. Using rapid point-of-care tests, 5.2% of earthquake survivors in the Southeastern Anatolia Region tested positive for HBsAg. Being male and having a family history of HBV are identified as significant risk factors. More extensive multicenter studies would be beneficial for detecting the prevalence and identifying specific risk factors for HBV and HCV infections in the general population.

## Supplementary Information

Below is the link to the electronic supplementary material.

Supplementary Material 1

## Data Availability

The datasets used and/or analyzed during the current study are available from the corresponding author upon reasonable request, but restrictions apply to their availability.

## References

[1] Mavrouli M, Mavroulis S, Lekkas E, Tsakris A. The impact of earthquakes on public health: a narrative review of infectious diseases in the post-disaster period aiming to disaster risk reduction, 2023. 10.3390/microorganisms11020419. 10.3390/microorganisms11020419 PMC9968131 36838384

[2] Chang Y, Zhang Y, Zhang H. Tectonic geomorphology of Türkiye and its insights into the neotectonic deformation of the Anatolian plate. Earthq Res Adv. 2024;4(1). 10.1016/j.eqrea.2023.100267.

[3] Picozzi M, Iaccarino AG, Spallarossa D. The preparatory process of the 2023 Mw 7.8 Türkiye earthquake. Sci Rep. 2023;13(1). 10.1038/s41598-023-45073-8. 10.1038/s41598-023-45073-8 PMC10587168 37857660

[4] WHO. WHO Türkiye earthquake: external situation report no. 9: 1 May–4 June 2023. https://iris.who.int/bitstream/handle/10665/370621/WHO-EURO-2023-7145-46911-70035-eng.pdf?sequence=1.

[5] Association TM. Türk Tabipleri Birliği VI. Ay Deprem Raporu. Accessed: Nov. 06, 2024. [Online]. Available: http://www.ttb.org.tr/userfiles/files/6ayraporu.pdf.

[6] Agency A. https://www.aa.com.tr/tr/dosya-haber/diyarbakirda-16-bin-466-depremzede-kira-yardimi-aliyor/2961136.

[7] Khan S, Rai MA, Khan A, Farooqui A, Kazmi SU, Ali SH. Prevalence of HCV and HIV infections in 2005-earthquake-affected areas of Pakistan. BMC Infect Dis. 2008;8. 10.1186/1471-2334-8-147. 10.1186/1471-2334-8-147 PMC2583978 18954443

[8] Tozun N, et al. Seroprevalence of hepatitis B and C virus infections and risk factors in Turkey: a fieldwork TURHEP study. Clin Microbiol Infect. 2015;21(11). 10.1016/j.cmi.2015.06.028. 10.1016/j.cmi.2015.06.028 26163105

[9] Tosun S, et al. 15-year evaluation of changes in the HBsAg positivity rate in pregnant women in Turkey: the prominent effect of national vaccination. East Mediterr Health J. 2022;28(10). 10.26719/emhj.22.071. 10.26719/emhj.22.071 36382732

[10] Dayan S, et al. HBsAg, anti-HCV, anti-HIV 1/2 and syphilis seroprevalence in healthy volunteer blood donors in southeastern Anatolia. J Infect Dev Ctries. 2013;7(9)665–9. 10.3855/jidc.2835. 10.3855/jidc.2835 24042102

[11] Dayan S, Özekinci T, Bekçibaşi M, Deveci Ö. HBsAg, anti-HCV and anti-HIV Seroprevalence among blood donors in southeastern Anatolia, Turkey, 2011–2015. Infez Med. 2019 Sep;27(3):316–21. https://www.infezmed.it/media/journal/Vol_27_3_2019_11.pdf. 31545776

[12] Idilman R, et al. A micro-elimination approach to addressing hepatitis C in Turkey. BMC Health Serv Res. 2020;20(1). 10.1186/s12913-020-5019-8. 10.1186/s12913-020-5019-8 PMC7093960 32209103

[13] World Health Organisation. Guidelines on hepatitis B and C testing, 66, no. February. 2017. https://iris.who.int/bitstream/handle/10665/254621/9789241549981-eng.pdf.

[14] Avellon A, et al. Clinical performance of determine HBsAg 2 rapid test for hepatitis B detection. J Med Virol. 2020;92(12). 10.1002/jmv.25862. 10.1002/jmv.25862 32270883

[15] Turkish Statistical Institute. http://data.tuik.gov.tr/Bulten/Index?p=Adrese-Dayali-Nufus-Kayit-Sistemi-Sonuclari-2023-49684. Accessed: Nov. 06, 2024. [Online]. Available: https://data.tuik.gov.tr/Bulten/Index?p=Adrese-Dayali-Nufus-Kayit-Sistemi-Sonuclari-2023-49684.

[16] Turhanoǧlu M, Onur A, Bilman FB, Ayaydin Z, Aktar GS. Eight-year seroprevalence of HBV, HCV and HIV in Diyarbakir training and research hospital. Int J Med Sci. 2013;10(11). 10.7150/ijms.6506. 10.7150/ijms.6506 PMC3775121 24046538

[17] Infectious-disease-tests TURKLAB. https://www.turklab.com.tr/infectious-disease-tests. Accessed: Nov. 06, 2024. [Online]. Available: https://www.turklab.com.tr/infectious-disease-tests.

[18] Duracinsky M, et al. Development of a risk prediction score for screening for HBV, HCV and HIV among migrants in France: results from a multicentre observational study (STRADA study), BMJ Open. 2024 Jun;14(6). 10.1136/bmjopen-2023-075315. 10.1136/bmjopen-2023-075315 PMC11163614 38839381

[19] Peeling RW, Holmes KK, Mabey D, Ronald A. Rapid tests for sexually transmitted infections (STIs): the way forward, 2006. 10.1136/sti.2006.024265. 10.1136/sti.2006.024265 PMC2563912 17151023

[20] Akcam FZ, Uskun E, Avsar K, Songur Y. Hepatitis B virus and hepatitis C virus Seroprevalence in rural areas of the Southwestern region of Turkey. Int J Infect Dis. 2009;13(2). 10.1016/j.ijid.2008.07.005. 10.1016/j.ijid.2008.07.005 18945630

[21] Mehmet D, Meliksah E, Serif Y, Gunay S, Tuncer Ö, Zeynep S. Prevalence of hepatitis B infection in the southeastern region of turkey: comparison of risk factors for HBV infection in rural and urban areas. Jpn J Infect Dis. 2005;58(1). 10.7883/yoken.jjid.2005.15. 15728984

[22] Ozkan H. Epidemiology of chronic hepatitis B in Turkey. Euroasian J Hepatogastroenterol. 2018;8(1). 10.5005/jp-journals-10018-1264. 10.5005/jp-journals-10018-1264 PMC6024053 29963468

[23] Tosun S. Viral hepatitlerin ülkemizdeki değişen epidemiyolojisi. Ankem Derg, 27, no. Suppl 2, 2013. https://ankemdernegi.org.tr/ANKEMJOURNALPDF/ANKEM_27_Ek2_128_134.pdf.

[24] Gower E, Estes C, Blach S, Razavi-Shearer K, Razavi H. Global epidemiology and genotype distribution of the hepatitis C virus infection, 2014. 10.1016/j.jhep.2014.07.027. 10.1016/j.jhep.2014.07.027 25086286

[25] Dursun M, et al. Prevalence of hepatitis C in adults in the South-eastern region of anatolia: A community-based study. Hepatol Res. 2004;29(2). 10.1016/j.hepres.2004.02.012. 10.1016/j.hepres.2004.02.012 15163428

