# Supplementary material for: Detection of HBV and HCV Seroprevalence using rapid point-of-care tests among earthquake survivors in Southeastern Türkiye
Source: BMC Infect Dis. 2025 Dec 24;25:1734. doi: 10.1186/s12879-025-12151-3 (PMC12729748; doi:10.1186/s12879-025-12151-3)
Supplement: Supplementary file 1 — Supplementary Material 1 [file 12879_2025_12151_MOESM1_ESM.docx]

**Viral Hepatitis Rapid Test Screening Form**

**Name- surname :**

**TR ID number :**

**Age:**

**Gender:** Male Female

**Education status:** None Primary school Secondary school

High school University

**Family history of hepatitis B:** Yes No

(Mother, father, sibling, uncle, maternal uncle, aunt, etc.)

**Marital status:**  Single Married

**Previous Surgery:** Yes No

**Dental treatment or surgery:** Yes No

**Tattoos or piercings:** Yes No

**Blood transfusion (1996 and before):**  Yes No

**Substance addiction (Marijuana, alcohol, intravenous drug ):** Yes No
